# Antagonistic Activity of Chilean Strains of *Pseudomonas protegens* Against Fungi Causing Crown and Root Rot of Wheat (*Triticum aestivum* L.)

**DOI:** 10.3389/fpls.2020.00951

**Published:** 2020-06-25

**Authors:** María Paz Castro Tapia, Ricardo P. Madariaga Burrows, Braulio Ruiz Sepúlveda, Marisol Vargas Concha, Carola Vera Palma, Ernesto A. Moya-Elizondo

**Affiliations:** ^1^ Departamento de Producción Vegetal, Facultad de Agronomía, Universidad de Concepción, Chillán, Chile; ^2^ National Agricultural Research Institute, INIA Quilamapu, Chillán, Chile

**Keywords:** Fusarium crown rot, take-all, sharp eyespot, *Pseudomonas protegens*, *Triticum aestivum*, 2,4-DAPG

## Abstract

Seed treatments with antagonistic bacteria could reduce the severity of crown and root rot diseases in wheat crops. The objective of this study was to evaluate the potential antagonistic activity of a bacterial consortium of three Chilean strains of *Pseudomonas protegens* against the wheat crown and root rot pathogens *Gaeumannomyces graminis* var. *tritici*, *Rhizoctonia cerealis*, and *Fusarium culmorum*. Two field experiments were carried out on artificially infested soil during two consecutive seasons (2016–2017 and 2017–2018) in an Andisol soil of southern Chile. Control treatments (not inoculated with fungi) were also included. Each treatment included a seed treatment of spring wheat cv. Pantera-INIA with and without the bacterial consortium. Both phytosanitary damage (incidence and severity) and agronomic components were evaluated. Bacterial populations with the *phlD*+ gene in the wheat plant rhizosphere during anthesis state (Z6) were also quantified. In both seasons, infection severity decreased by an average of 16.8% in seeds treated with *P. protegens* consortium, while yield components such as spikes m^−1^ and number of grains per spike increased. The use of antagonistic bacteria resulted in a total yield increase only during the first experimental season (P < 0.05). In general, accumulated rainfall influenced the antagonistic effect of the consortium of *P. protegens* strains, accounting for the differences observed between the two seasons. The results suggest that this *P. protegens* consortium applied on seeds can promote plant growth and protect wheat crops against crown and root rot pathogens in Southern Chile under field conditions.

## Introduction

Wheat cultivation (*Triticum aestivum*) is severely affected by several soil-borne pathogenic fungi that cause diseases of economic importance. Pathogens associated with root and crown rot of wheat plants include species of the genera *Gaeumannomyces, Fusarium*, and *Rhizoctonia*. Crown and root rot diseases affect the vascular system and hence interfere with the normal transport of water and nutrients. Severe infections lead to death of the plant and can result in significant economic losses (Daval et al., 2010). In southern Chile, several species of phytopathogenic *Fusarium* and *Rhizoctonia* have been isolated from commercial wheat crops (Moya-Elizondo et al., 2015), whereas *Gaeumannomyces graminis* var. *tritici* has been identified as the phytopathogenic fungus that causes the greatest losses in wheat yields in the country (Andrade et al., 2011; Vera et al., 2014).

*Gaeumannomyces graminis* var. *tritici* causes take-all disease of cereals, which damages roots, crown, and culms of the plant and strongly reduces crop yield (McMillan et al., 2014; Vera et al., 2014), mainly surviving as mycelium in a saprophytic manner in culms, crowns, and roots of wheat plants and other susceptible grasses. This fungus produces runner hyphae, which grow superficially and then cover and penetrate tissues in the root area, colonizing and destroying vascular tissues, thus interfering the transport of water and nutrients in the host plant (Kwak and Weller, 2013; Quan et al., 2015). As a consequence, wheat plants develop chlorosis, and/or present stunted crop growth, formation of white spikes during flowering, reduced seed formation or plant death. The genetic variability of *G. graminis* var. *tritici* identifies two genetic groups capable of co-existing, but with differences in the severity of infection under wheat monoculture as well as different sensitivity to fungicides (Lebreton et al., 2004; Daval et al., 2010). A third genetic group, also capable of causing severe infections in wheat plants, was identified by using RAPD (random amplification polymorphism DNA) fingerprinting while sampling 48 commercial wheat fields in the 2011–2012 season in the Araucanía, Los Ríos, and Los Lagos regions of Chile (unpublished data).

The fungal complex of the genus *Fusarium* is composed of several species that affect cereal crops, including *F*. *graminearum* Schwabe, *F. culmorum* (Smith) Sacc., and *F. pseudograminearum* Aoki and O’Donnell, which has been particularly associated with severe crown and root rot disease of wheat (Pecoraro et al., 2018; Moya-Elizondo et al., 2011). These species have been reported as significant pathogens in wheat cultivation worldwide, affecting both final yield and grain quality (Scherm et al., 2013). Moreover, members of the genus *Fusarium* are capable of producing mycotoxins such as deoxynivalenol (DON) and zearalenone (ZEA), which are secondary metabolites that can contaminate grains, posing risks to human and animal health (Rohweder et al., 2011).

In wheat, the species *R. solani* Kuhn (AG 8), *R. oryzae* Ryker and Gooch, and *R. cerealis* E. P. Hoeven are important soil-borne pathogens (Mavrodi et al., 2012b; Weller et al., 2016). Members of the *Rhizoctonia* complex attack roots, and penetrate into and decay the culm, affecting plant vigor and wheat production (Okubara et al., 2014). In Chile, the three abovementioned species have been identified as pathogens affecting wheat crops (Moya-Elizondo et al., 2015; Doussoulin et al., 2016). However, there is little information known regarding the quantitative damage caused by these Rhizoctonia fungi in wheat grown in southern Chile.

Crown and root rot diseases are difficult to control. Their management involves the integration of cultural and genetic, as well as chemical and biological control methods (Scherm et al., 2013; Weller, 2015). At present, there are no resistant *G. graminis* var. *tritici* wheat varieties available, and although chemical control has had some success, the disease is not completely controlled (Vera et al., 2014). In this context, biological control seems to be a sustainable alternative for the management of fungal soil diseases, while suppressive soils could be an important source of biocontrol agents to manage root rot diseases of wheat (Raaijmakers et al., 2009; Doussoulin and Moya-Elizondo, 2011; Weller, 2015; Expósito et al., 2017; Durán et al., 2018). In suppressive soils, bacteria of the genus *Pseudomonas* have been described as antagonistic agents against pathogens, because they produce antibiotics, such as 2,4-diacetylphloroglucinol (2,4-DAPG), phenazine-1-carboxylic acid (PCA), pyrrolnitrin, rhizoxin, pyoluteorin, cyanide hydrogen (HCN), and 2-hexyl-5-propyl resorcinol (HPR) (Ramette et al., 2011; Loper et al., 2012). Among the groups of pseudomonads species, *Pseudomonas protegens* are characterized by 2,4-DAPG, pyrrolnitrin, and pyoluteorin production. This bacterium has recently been detected in commercial wheat crops in Chile (Moya-Elizondo et al., 2013).

Recent studies in Chile have evaluated the effect of *P. protegens* on take-all disease (Moya-Elizondo et al., 2013; González et al., 2018; Vera et al., 2019). However, the extent to which a consortium of different isolates of this antagonistic bacterium can reduce the damage caused by different crown and root rot fungi on wheat has not been determined. Therefore, the objective of this study was to evaluate the potential antagonistic activity of a consortium of three Chilean *Pseudomonas protegens* strains against three phytopathogenic fungi that cause crown and root rot diseases in wheat in an Andisol soil in southern Chile. Field trials with artificial inoculation of three crown and root rot pathogens of wheat and a seed treatment with a consortium of *P. protegens* strains were conducted. Both phytosanitary and agronomic parameters were evaluated. The former included incidence and severity of root and crown rot disease, and the latter included height (cm), number of grains, spikes and grains/spike m^−1^, weight of one thousand grains (g), hectoliter weight (kg hl^−1^), biomass accumulation m^−1^ (g), and yield (ton ha^−1^). In addition, the population dynamics of the bacteria on the wheat rhizosphere was studied.

## Materials and Methods

### Study Area

Two field experiments were carried out in two consecutive seasons, 2016–2017 (season 1) and 2017–2018 (season 2), in the Santa Rosa Experimental Station at the Quilamapu Regional Research Center of the National Agricultural Research Institute (INIA), Chillán, Ñuble Region, Chile (36°31’53’’ S, 71°54’50.1’’ O, 220 m.a.s.l). The soil corresponds to an Andisol (Typic Melanoxerands) of the Arrayán series that uses gravitational irrigation (Stolpe, 2006). The experiments were seeded with spring wheat (*T. aestivum*) cv. Pantera-INIA and established in a completely randomized block design with 10 treatments and four replicates (**Table 1**). Each treatment included artificial soil inoculation with one of the four wheat pathogenic fungi under study and a control treatment not inoculated (CNIF). Each one of the treatments, including the control, was either seeded with seeds treated or untreated with a consortium of three strains of *P. protegens* (+Pp or −Pp, respectively). Seeding was conducted on August 8, 2016 and August 16, 2017 in season 1 and 2, respectively. The experimental unit consisted of a plot with six rows, 2 m long and spaced 0.2 m apart (2 m^2^). In the case of rows inoculated with phytopathogenic fungi, oat grains inoculated with the pathogens were placed next to the wheat seeds at sowing time at a 1:3 ratio. Only the four central rows of each plot were inoculated.

**Table 1:** Treatments used to evaluate a seed treatment with a consortium of three *Pseudomonas protegens* (Pp) strains against crown and root rot pathogens (artificially inoculated) in field experiments with spring wheat cv. Pantera-INIA.

Treatment code	Fungus inoculated	Isolate name	*P. protegens* Factor	Inoculum factor
CNIF + Pp	Control not inoculated with fungi	–	+	−
CNIF − Pp			−	−
GGT2 + Pp	*G. graminis* var. *tritici* group 2	2010_04_G ^a^	+	+
GGT2 − Pp			−	+
GGT3 + Pp	*G. graminis* var. *tritici* group 3	Oso1 ^b^	+	+
GGT3 − Pp			−	+
Fc + Pp	*Fusarium culmorum*	F_CULM ^c^	+	+
Fc − Pp			−	+
Rc + Pp	*Rhizoctonia cerealis*	M31S ^c^	+	+
Rc − Pp			−	+

Isolates are described for the following authors: ^a^Vera et al. (2014; 2019), ^b^ González et al. (2018), and ^c^ Moya-Elizondo et al. (2015).

### Phytopathogenic Fungi Inoculum Preparation

The species of fungi used in the study were highly pathogenic *G. graminis* var. *tritici* isolate 2010_04_G (GGT2) belonging to the genetic group 2 (Vera et al., 2014; Vera et al., 2019), *G. graminis* var. *tritici* isolate Oso1 belonging to the proposed new genetic group 3 (GGT3) (unpublished data), *F. culmorum* isolate F_CULM (Fc), and *R. cerealis* isolate M31S (Rc). The fungal isolates were collected from commercial wheat crop fields in southern Chile (Moya-Elizondo et al., 2015). Fungi were maintained in the Plant Pathology Laboratory of the Universidad de Concepción, Campus Chillán, Chile, and grown in PDA medium during 7 d at 24 ± 2°C prior to inoculum preparation.

The inoculum preparation for all phytopathogenic fungi was carried out according to the methodology described by Vera et al. (2014). Briefly, 500 ml Erlenmeyer flasks were filled with 200 g of oat and 100 ml of distilled water, soaked for 24 h, and autoclaved for two consecutive days at 120°C for 15 min, at a pressure of 15 psi. Subsequently, 20 pieces of agar from each individual pathogenic isolate were cut and placed to the sterile flasks with oat, and maintained during 15 d for *Fusarium* and *Rhizoctonia* isolates and 30 d for *G. graminis* var. *tritici* isolates to ensure that the kernels could be uniformly colonized by each pathogen. During colonization time, the flasks with inoculated oat kernels were shaken on daily basis until use. The oat kernels were then sown together with the wheat seeds in the field as described above.

### Seed Treatment With a Consortium of *Pseudomonas protegens* Strains

A bacterial consortium composed of *P. protegens* strains Ca10, Ca6, and ChB7 was used to treat the wheat seeds. The bacteria were isolated from the wheat rhizosphere from different Andisol soils located in southern Chile (Moya-Elizondo et al., 2013). ChB7 strain was obtained from a wheat field located in Chillán, Ñuble Region (36°35’55’’S 72°04’42’’W); whereas strains Ca10A and Ca6 were isolated from two wheat fields located in Cajón, a locality 8 km to the north of Temuco, Araucanía Region, Chile (38°40’04’’S 72°29’53’’W). Analyses performed in the laboratory showed the presence of *phlD*, *plt*, and *prn* genes, which are associated with 2,4-DAPG, pyoluteorin, and pyrrolnitrin production in these three bacterial strains. Moreover, the strains belong to the genetic group A of the *phlD* gene and have proven efficient to promote growth through phosphorus (P) solubilizing activity and IAA (indole acetic acid) production (unpublished data). These bacteria were stored at −80°C in King’B medium plus 20% glycerol, at the Plant Pathology Laboratory of the Universidad de Concepción, Chillán, Ñuble Region, Chile.

In order to obtain the bacterial concentration, a volume of 40 μl of each stored strain was individually placed into Falcon tubes with 10 ml of KB broth, and kept in agitation at 150 rpm during 24 h. Eight ml were transferred to a flask with 200 ml of KB broth, which was subsequently shaken using a rotary shaker at 250 rpm during 48 h. Bacterial suspensions of the Ca10, Ca6, and ChB7 strains [~10^10^ colony forming units (CFU) ml^−1^] were centrifuged at 4,000 rpm at 20°C for 8 min. The supernatant was eliminated and the samples were washed in a buffer (25 ml sodium chloride 0.9%) to be centrifuged again under the same conditions for 5 min and the supernatant was removed. Total volumes of 100 µl sterile KB medium and 3 ml of 0.5% carboxymethyl cellulose were added to improve bacterial adherence to the seeds. Finally, a volume of 3.1 ml of bacterial suspension (~10^8^ CFU g^−1^ of seed) was mixed at a vortex and then applied to 1 kg of seeds before seeding. Seed were dried under sterile conditions in a laminar flow cabinet during 6 h before seeding. A dose of 50 g of seeds per experimental plot was used for each treatment with (+Pp) and without (−Pp) the antagonistic bacteria.

### Agronomic Management and Weather Conditions

Once the soil was prepared and herbicide applied, seeding was carried out manually. The products used were flufenacet+flurtamone+diflufenican (Bacara^®^ Forte 360 SC, Bayer S.A.). A second application of herbicide was performed at tillering, growth stage Z2.3 (Zadoks and Schein, 1974), using iodosulfuron-methyl-sodium + mesosulfuron-methyl (Cossack 150 WG, Bayer S.A.). Fertilization broadcast was carried out at seeding, with DAP (diammonium phosphate, 260 kg ha^−1^), potassium chloride (KCl) (60 kg ha^−1^), Sulpomag (200 kg ha^−1^), boronatrocalcite (10 kg ha^−1^), and Zn sulfate (ZnSO_4_) (3 kg ha^−1^). Nitrogen fertilization was split-applied by using 133 units of sodium nitrate (NaNO_3_): 93 units (375 kg ha^−1^) in main shoot and three tillers state (Z2.3 Zadoks scale) and 60 units (580 kg ha^−1^) at the end of the tillering state (Z2.9 Zadoks scale).

**Figure 1:**
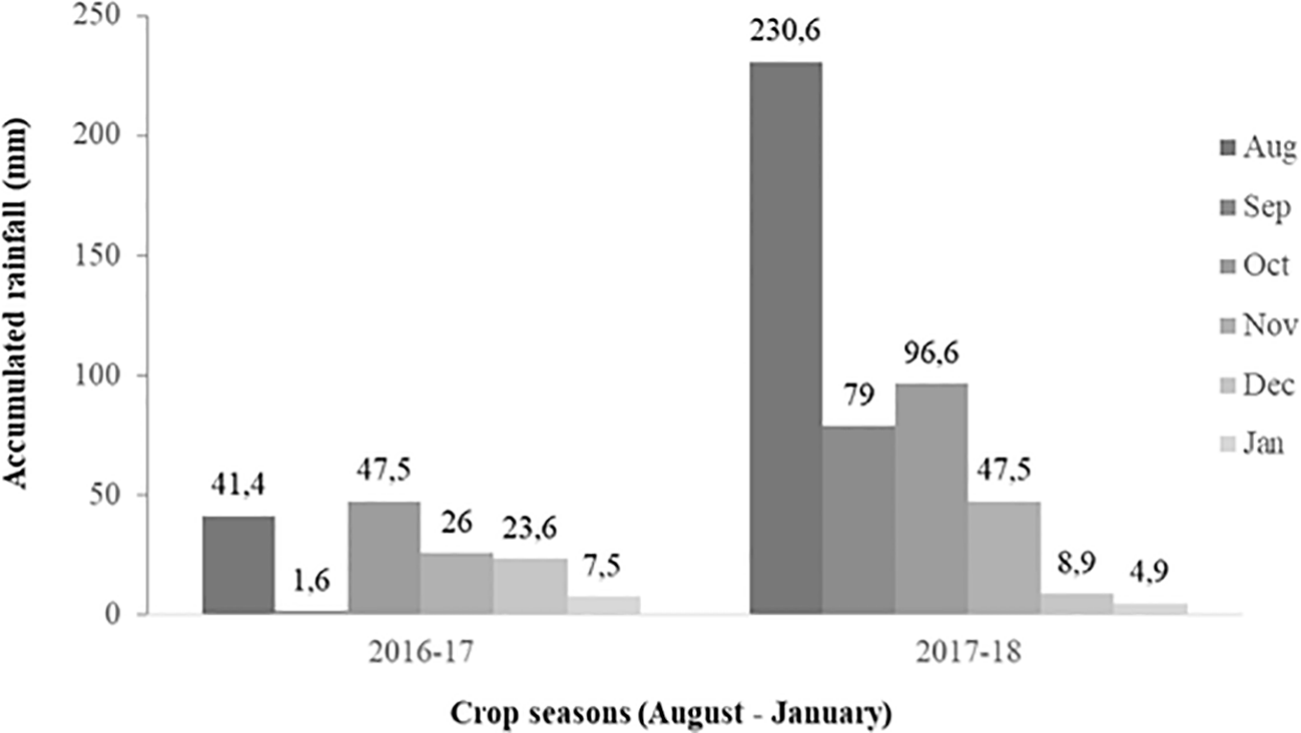
Monthly accumulated rainfall (mm) in Chillán, Ñuble Region, Chile, between August (seeding) to January (harvest) of season 1 (2016–2017) and 2 (2017–2018).

The average monthly rainfall was 24.6 and 77.9 cm in season 1 and season 2, respectively (**Figure 1**). Regarding irrigation, plots were flood irrigated twice in season 1 (October 11 and November 9, 2016), and once in season 2 (November 21, 2017). The average monthly air temperature was quite similar in both seasons (14°C in both crop cycles). Climatic information was obtained from the agrometeorological station available at the INIA Quilamapu Santa Rosa Experimental Station.

### Determination of Bacterial Populations of *Pseudomonas protegens* Present in the Seed Before and After Seeding and in the Rhizosphere of Wheat Plants in Anthesis (Z6)

Bacterial populations of *P. protegens* in the wheat seeds were quantified after seeds were treated (prior to seeding) and 24 h later since this was the time period between seed inoculation and seeding in the field. Determination of initial bacterial inoculum in seeds was performed through serial dilutions, where a gram of seeds inoculated with the bacterial consortium was placed in 9 ml of sterile distilled water (SDW) and vortexed for 25 s. Then, 100 µl of each serial dilution were grown in KB agar medium. Dishes were incubated at 25°C for 24 h to determine CFU g^−1^ seeds. A random sample of approximately 10 g of root tissue from each plot was collected in the anthesis state (Z6). Roots were cleaned by removing the excess soil with sterile brushes. Samples were placed in 15 ml individual centrifuge tubes and stored at −80°C until use. Both quantification and bacterial detection of the samples collected in anthesis were carried out using the real-time quantitative PCR technique (qPCR), based on the protocols described by Vera et al. (2019). Briefly, 1 g of root tissue was collected from each plot and 9 ml SDW were added; the mixture was incubated for 24 h at room temperature. Samples were then vortexed four times for 25 s to remove bacteria from roots. DNA extraction was performed using the PowerSoil^®^ DNA Isolation kit (Qiagen N.V, Germany), following the instructions of the manufacturer. Standard curves with DNA purified from rhizospheres were created based on the protocol and parameters described by Mavrodi et al. (2007). Subsequently, an analysis of the presence of the *phlD* gene group A was also performed. This is associated with the fact that *P. protegens* strains ChB7, Ca10, and Ca6 belong to genetic group A of the *phlD* gene (unpublished data). StepOnePlus™ Real Time PCR Systems (Applied Biosystems) equipment was used. For the analysis, primers A_Up and A_Low (Mavrodi et al., 2007) were used, and each qPCR reaction contained a mixture of 5 µl SYBR^®^ Green (KAPA SYBR^®^ FAST qPCR, KAPA Biosystems), 2 µl of ADN, 0.1 µM ROX (passive reference), and 0.5 µM of each primer until completing a final volume of 10 µl with ultrapure sterile water. The qPCR plate was loaded with three technical replicates of each sample.

### Evaluation of Disease Damage

All plants in each experimental plot were inspected visually on a weekly basis in order to detect symptoms of root rot disease. Once the first symptoms were observed, the damaged area presenting chlorotic patches, stunted growth and presence of white spikes was estimated. Data obtained were used to calculate the area under the disease progress curve (AUDPC), which combines multiple observations of visual symptoms over time and expresses them into one single value (Simko and Piepho, 2012).

Crown and root rot incidence was assessed in each experiment by sampling the plants within an area of 50 linear cm of the second row of each plot. Wheat plants were collected 1 d before harvest and disease incidence in each plot was determined as the percentage of symptomatic crowns and roots in a culm from the total number of assessed culms (80 culms). In addition, disease severity or IDSI (internodes discoloration severity index) was determined through a 5-digit visual scale, corresponding to the percentage of damage observed in the first internode of the wheat culm (class value), where 0 = 0% or without symptoms, 1 = 1–25%, 2 = 25–50%, 3 = 50–75%, and 4 = 75–100% of darkening of the tissue in the first internode (Moya-Elizondo et al., 2015). The IDSI for each field was then calculated as: [Σ (class value × frequency)/(total number of culms × the highest class value)] × 100 (Hogg et al., 2007), and it was evaluated in 80 culms of wheat plants 1 d after harvest. In addition, a sample of 10 symptomatic culms was taken for the re-isolation of pathogens from each experimental plot.

### Plant Development and Agronomic Parameters Evaluated

Ten days after seeding, plant emergence was evaluated by counting the number of plants present in an area of one linear meter within the four central rows. After emergence, phenological crop stages were determined on a weekly basis using the Zadoks scale (Zadoks and Schein, 1974). Similarly, plant height was also recorded weekly until harvest maturity (Z9).

One day prior to harvest, wheat plant samples were collected to determine the number of spikes per m^2^, number of grains per spike, and thousand grain weight. The sample in each experimental plot consisted of 20 spikes collected within an area of one linear meter of the second row, which were then manually threshed to obtain the kernels (80 spikes per each treatment). In addition, total biomass and harvest index (HI) were determined. The HI is the ratio of harvested grain to total shoot dry matter, and this can be used as a measure of reproductive efficiency. The harvest was carried out on January 23, 2017 and January 26, 2018 in season 1 and 2, respectively, using a Wintersteiger threshing machine and considering only the four central rows of each experimental plot. Wheat test weight (kg hl^1^) and grain yield per plot (ton ha^−1^) were calculated.

### Statistical Analysis

The experiments were established in a completely randomized block design. The results of each evaluation were subjected to an analysis of variance (ANOVA), after checking the assumptions of normality, homoscedasticity, and independence of the data. When significant differences were found, Fisher’s least significant difference (LSD) method was used to determine differences between the treatments (p < 0.05). Percentages were adjusted through the square root transformation through the formula y = √(x + 0.5), where x = percentage value to be transformed (Little and Hills, 1978). Pearson correlation analyses were performed to determine the relations between pathogenicity fungal isolates and bacterial populations of *P. protegens* with respect to the variables evaluated. All analyses were performed using SAS software Version 8 (SAS Institute Inc., 1999).

## Results

### Quantification of Populations of *P. protegens* in Seed and Rhizosphere of Wheat Plants

**Figure 2:**
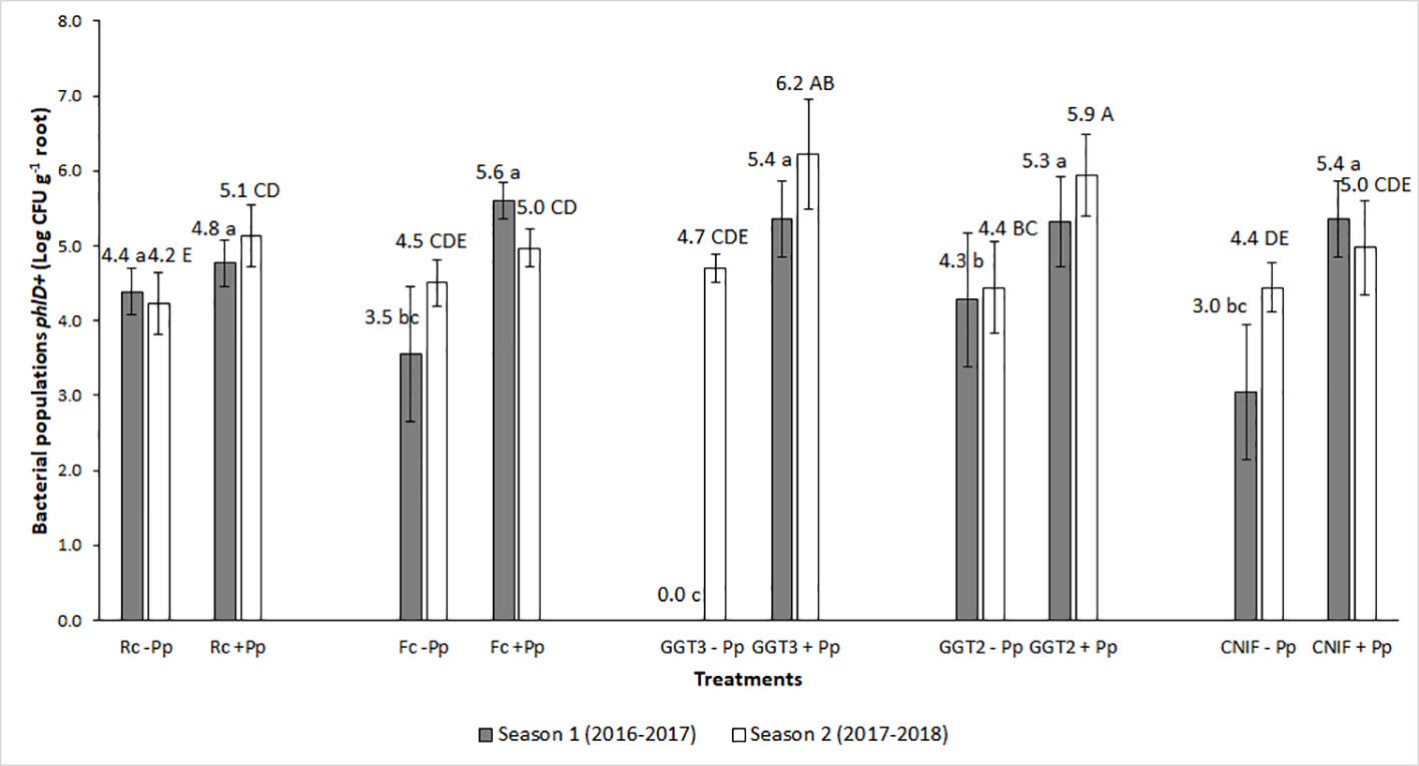
Bacterial populations *phlD+* gene (Log CFU per g of root) obtained by qPCR from wheat roots of cv. Pantera-INIA at anthesis stage (Z.6) in treatments of seeds treated with or without a consortium of *Pseudomonas protegens* (+ Pp + or − Pp, respectively) and inoculated with different crown and root rot pathogens (two isolates of *Gaeumannomyces graminis* var. *tritici* [GGT], *Fusarium culmorum* (Fc), and *R. cerealis* (Rc) in two crop seasons. NCIF, Control not inoculated with fungi. Different lowercase letters on bars indicate significant differences between treatments during the 2016–2017 season, while different capital letters indicate differences between treatments in the 2017–2018 season, according to mean comparison LSD test (α = 0.05). Description of the treatments is available in **Table 1**.

Pre- and post- seeding quantification of the bacterial inoculum on seeds ranged between 10^9^ and 10^7^ CFU g^−1^ of seed (data not shown). For both seasons, bacterial populations in the rhizosphere of wheat plants during the phenological state of anthesis (Z6) showed differences between the treatments with (+Pp) and without (−Pp) application of the *P. protegens* consortium (P < 0.05; **Figure 2**). Inoculation of the bacterial consortium increased the populations of bacteria *phlD*+ in the rhizosphere, moving from an average of Log 3.05 CFU g^−1^ of root to Log 5.28 CFU g^−1^ root in season 1, and from Log 4.46 CFU g^−1^ to 5.45 CFU g^−1^ in season 2 for untreated and treated seeds with the bacterial consortium of *P. protegens*, respectively (**Figure 2**).

The highest responses in bacterial *phlD*+ population in roots for the seed treatment with *P. protegens* (+Pp) were observed in those plots inoculated with the two isolates of *G. graminis* var. *tritici* (GGT2: isolate 2010_04_G; and GGT3: isolate Oso1), reaching more than Log 4.3 CFU g^−1^ of root in both seasons (**Figure 2**). Conversely, these bacterial populations were not detected in the roots of the treatment *G. graminis* var. *tritici* isolate Oso1 without inoculation with *P. protegens* (GGT3 −Pp) during anthesis in season 1 (**Figure 2**). This occurred because their concentration in the sample was below the detection range of the qPCR protocol used to estimate their presence. Infection associated with inoculation with *F. culmorum* and *R. cerealis* did not increase the population of *phlD*+ bacteria with respect to the control (CNIF −Pp) or seed not treated with *P. protegens* (Fc −Pp and Rc −Pp treatments). Seed treatment with the consortium of *P. protegens* increased the bacterial populations in wheat roots in those plots inoculated with *F. culmorum* (Fc +Pp) and *R. cerealis* (Rc +Pp). However, this variation was variable and did not differ from the controls [CNIF +Pp and CNIF −Pp in some experiments (**Figure 2**)].

### Effect of a *Pseudomonas protegens* Consortium on Disease Symptoms Caused by Crown and Root Rot Pathogens in Wheat

Disease severity progress (percentage of leaf area covered with symptoms) was expressed as AUDPC values, which integrated all visual assessments conducted on weekly basis in the experimental plots (**Table 2**). In season 1, significant differences were found between the plots with and without inoculation of *P. protegens* in all the inoculated fungi, with the exception of those inoculated with *F. culmorum* (**Table 2**). The control CNIF +Pp recorded the lowest AUDPC values and was the healthiest of all the treatments assessed in the season, recording an AUDPC value of 1237, while *G. graminis* var. *tritici* isolate 2010_04_G (GGT2) showed the highest AUDPC value in this experiment. No differences were found between the controls (CNIF +Pp and CNIF −Pp) and the treatments inoculated with *F. culmorum* with or without seed treatment of the *P. protegens* consortium (Fc +Pp and Fc −Pp), while these treatments showed no differences between them and recorded the lowest AUDPC values. An increase in the plot area covered with symptoms was observed in the *R. cerealis* and *G. graminis* (GGT3 and GGT2) treatments where seeds were not treated with the bacterial consortium (**Table 2**).

**Table 2:** Area under the disease progress curve (AUDPC) values on foliage, percentage of incidence and crown and root rot severity index (IDSI)^a^ observed in treatments of wheat cv. Pantera-INIA seed treated with (+ Pp) and without (– Pp) a consortium of three *Pseudomonas protegens* strains against crown and root rot pathogens (artificially inoculated) in two consecutive crop seasons (including two control treatments (CNIF) with no fungal inoculation).

	Season 1 (2016–2017)						Season 2 (2017–2018)		
Treatments ^b^	AUDPC		Incidence (%)		IDSI		AUDPC	Incidence (%)	IDSI
CNIF + Pp	1,237 e		35.0 bc		8.0 d		800 f	13.1 d	3.1 c
CNIF − Pp	1,633 cd		34.3 bc		8.6 d		1041 e	30.9 c	4.8 c
GGT2 + Pp	2,427 b		64.0 a		34.0 a		2065 a	68.5 a	34.5 a
GGT2 − Pp	3,042 a		57.8 a		29.8 a		1967 ab	54.7 b	21.3 b
GGT3 + Pp	1,970 c		44.4 abc		15.8 bc		1801 bc	51.3 b	18.1 b
GGT3 − Pp	2,500 b		49.3 abc		16.8 b		1593 d	45.9 b	17.6 b
Fc + Pp	1,522 de		53.1 ab		13.6 bcd		1004 e	28.7 c	4.0 c
Fc − Pp	1,618 cd		34.3 c		10.5 cd		982 ef	21.3 cd	4.3 c
Rc + Pp	1,945 c		32.8 c		8.3 d		1149 e	32.2 c	6.5 c
Rc − Pp	2,407 b		29.3 c		7.1 d		1618 cd	32.6 c	5.9 c
**CV ^c^**	12.0		18.2		20.12		9.65	9.49	22.4
**P value ^d^**	< 0.0001		0.0164		< 0.0001		< 0.0001	< 0.0001	< 0.0001

a IDSI, internodes discoloration severity index, where the scale included six classes (0–5), where 0 = no infected internode, 1 = < 25% of infected internode, 2 = 25%–50% of infected internode, 3 = 50%–75% of infected internode, 4 = 75%–100% of infected internode, and 5 = > 100% or infection in upper internodes. The IDSI for each plot was then calculated as: IDSI = [Σ (class value × frequency)/(total number of plants × the highest class value)] × 100 (Hogg et al., 2007), and it was evaluated in 80 stems of wheat plants 1 day after harvest. ^b^Description of the treatments is available in **Table 1**. ^c^CV, coefficient of variation. ^d^Different letters in the same column show significant differences between treatments according to the LSD comparison mean test (α = 0.05).

Incidence of culm tissue damage showed no significant differences between the seed treatments treated and untreated with the bacterial consortium for the control with no fungal inoculations and all the phytopathogens assessed, with the exception of *F. culmorum*, where the seed treatment with *P. protegens* strains increased necrosis of the culms (**Table 2**). In general, the seed treatments inoculated with the bacterial consortium did not affect damage incidence observed in the culms.

An internode discoloration severity index (IDSI) was calculated to determine the severity of infection observed in the first internode of the wheat plants. The IDSI presented no differences between the treatments Pp+ and −Pp for each one of the fungal pathogens inoculated. Values showed that both controls (CNIF Pp+ and CNIF −Pp), and the treatments inoculated with *R. cerealis* had the lowest infection rates (**Table 2**). Fungal artificial inoculation showed that *R. cerealis* and *F. culmorum* recorded mild to moderate infections below 14% in the first internode and roots, whereas GGT2 showed the highest severity of infection (29–34%), and severity of GGT3 remained under 17% in wheat plants. Moreover, a positive significant correlation was observed between the AUDPC values and the severity of infection observed in the first internode of the culms in season 1 (Pearson: 0.30; p = 0.0412).

In season 2, AUDPC values for the CNIF and Rc consortium seed treated (+Pp) treatments differed from those seeds not treated with the bacterial consortium (−Pp). In fact, the bacterial treatment resulted in a reduction of 30% and 40.7% in AUDPC values, respectively (P< 0.001; **Table 2**). GGT3 +Pp showed an AUDPC value 12% higher than GGT3 −Pp, whereas those treatments inoculated with GGT2 and *F. culmorum* presented no significant differences in the AUDPC value when seeds were either treated or not treated with the bacterial consortium (P< 0.001; **Table 2**). Both controls (CNIF +Pp and CNIF −Pp) as well as the treatments inoculated with *F. culmorum* and *R. cerealis* and seeds treated with *P. protegens* consortium (Fc +Pp and Rc +Pp, respectively) showed the lowest symptoms on wheat foliage. On the other hand, both *G. graminis* var *tritici* genotypes (+Pp or −Pp) and *R. cerealis* −Pp presented the highest observable damage symptoms on foliage, reaching an average AUDPC value of 1,808, which was 96.3% higher than the average of both controls (CNIF +Pp and CNIF −Pp). Incidence of damaged culms followed an infection pattern and control response on pathogens similar to that observed for AUDPC, where no difference were found between +Pp and −Pp treatments, except for CNIF and GGT2. The use of bacteria on the seeds markedly reduced the incidence of damage in the culms when comparing the two controls not inoculated with a fungus (135% less). Moreover, GGT2 +Pp had a greater damage in the culms than GGT2 −Pp (p < 0.0001). Disease progress observed in the first internode, expressed as a discoloration severity index (IDSI), showed no differences between +Pp and −Pp for both fungal inoculated and control treatments, except for the treatments inoculated with GGT2 since GGT2 +Pp resulted in an increased damage and was significantly different from its counterpart GGT2 −Pp.

### Effect of *Pseudomonas protegens* Consortium on Agronomic Variables in Wheat Inoculated With Crown and Root Rot Pathogens

Final plant height per treatment (season 1 and 2) is shown in **Table 3**. In season 1, a significant difference was found in plant height between +Pp and −Pp for the treatments inoculated with GGT3 and *F. culmorum* (p = 0.018). However, seed treatment with consortium of *P. protegens* showed higher average plant height; these differences were not significant for CNIF, GGT2, and Rc in season 1. In general, inoculation with the pathogens markedly reduced plant growth, although GGT2 −Pp and GGT3 −Pp treatments showed the lowest growth rate at the end of the season. On the other hand, the bacterial seed treatment in season 2 did not show differences in plant height when each pathogen or CNIF was compared, although the treatments that reached the highest final plant growth were CNIF +Pp and Rc +Pp (≥94 cm in plant height). Those treatments inoculated with GGT2 and GGT3 reached the lowest plant height (88.2 cm in average). In both seasons, a negative correlation was observed between final plant height and AUDPC values (Pearson: −0.64, p <0.0001 and Pearson: −0.52, p = 0.0006, respectively).

**Table 3:** Final average plant height (cm) observed in treatments of wheat cv. Pantera-INIA seed treated with (+ Pp) and without (– Pp) a consortium of three *Pseudomonas protegens* strains against crown and root rot pathogens (artificially inoculated) in two consecutive crop seasons (including 2 control treatments (CNIF) with no fungal inoculation).

	Final plant height (cm)	
Treatments ^a^	Season 1(2016–2017)	Season 2 (2017–2018)
CNIF + Pp	91.8 ab	94.0 a
CNIF − Pp	88.5 bcde	91.2 abcd
GGT2 + Pp	88.8 bcde	87.7 cd
GGT2 − Pp	85.5 de	88.7 bcd
GGT3 + Pp	90.5 abcd	87.5 d
GGT3 − Pp	85.3 e	88.7 bcd
Fc + Pp	94.3 a	92.7 abcd
Fc − Pp	86.5 cde	91.7 abc
Rc + Pp	90.8 abcd	94.2 a
Rc − Pp	87.0 bcde	91.5 abcd
**CV ^b^**	3.86	3.14
**P value ^c^**	0.018	0.014

a Description of the treatments is available in **Table 1**. ^b^CV, coefficient of variation. ^c^Different letters in the same column showed significant differences among treatments according to the LSD comparison mean test (α = 0.05).

In season 1, the number of stems, spikes and grains/spike per a linear meter of the plots presented significant differences between the treatments (P <0.05; **Table 4**). In general, seed treatment with the bacterial consortium improved some of these parameters, especially when the three strains of *P. protegens* were adhered on the seeds in the control (CNIF +Pp), which recorded increases of 24.6% and 9.5% in spikes and grains/spike, respectively. The use of the bacterial consortium in this treatment also resulted in an increase in the number of stems (14.8%), but with no significant differences between both controls. Infection of GGT2 and GGT3 inoculation showed a marked reduction of the three parameters abovementioned. However, GGT3 +Pp resulted in a significant increase of 37 stems m^−1^, and an average of 32 spikes m^−1^. A similar effect was observed in GGT2 +Pp as the addition of the bacterial consortium resulted in an increase of 5 grains/spike m^−1^ more compared to GGT2 −Pp. In addition, significant differences were observed between GGT2 +Pp and GGT2 −Pp (p <0.05). Inoculation with *F. culmorum* and *R. cerealis* showed almost no differences with the controls (CNIF +Pp and CNIF −Pp) in terms of number of stems and spikes nor when these fungal treatments were seed treated with the antagonistic bacteria. However, regarding grains/spike m^−1^, plots inoculated with *F. culmorum* and *R. cerealis* had higher values than the control CNIF −Pp. Moreover, Fc +Pp and Fc −Pp differed significantly from both controls (CNIF +Pp and CNIF −Pp).

**Table 4:** Average number of stems and spikes m^−1^ and number of grains/spikes observed in treatments of wheat cv. Pantera-INIA seed treated with (+ Pp) and without (– Pp) a consortium of three *Pseudomonas protegens* strains against crown and root rot pathogens (artificially inoculated) in two consecutive crop seasons (including 2 control treatments (CNIF) with no fungal inoculation).

	Season 1(2016–2017)			Season 2 (2017–2018)		
Treatments ^a^	N° stems/m^−1^	N° spikes/m^−1^	N° grains/spikes	N° stems/m^−1^	N° spikes/m^−1^	N° grains/spikes
CNIF + Pp	122 ab	122 a	42 cd	112 a	109 a	50 n.s
CNIF − Pp	104 bc	92 b	38 e	109 abc	107 ab	47
GGT2 + Pp	111 abc	93 b	44 abc	87 d	87 cd	51
GGT2 − Pp	94 cd	90 b	39 e	72 e	72 d	53
GGT3 + Pp	116 abc	111 ab	40 de	97 bcd	95 abc	52
GGT3 − Pp	79 d	79 ab	41 de	89 d	87 cd	52
Fc + Pp	127 a	124 a	46 a	111 ab	106 ab	49
Fc − Pp	118 ab	118 a	46 ab	97 bcd	96 abc	51
Rc + Pp	122 ab	118 a	43 sd	95 cd	93 bcd	50
Rc − Pp	114 abc	111 ab	43 cd	95 cd	91 cd	50
**CV ^b^**	14.4	14.7	4.6	10.5	10.0	7.9
**P value ^c^**	0.009	0.026	< 0.0001	0.0003	0.003	0.600

a Description of the treatments is available in **Table 1**. ^b^CV, coefficient of variation. ^c^Different letters in the same column showed significant differences among treatments according to the LSD comparison mean test (α = 0.05).

In season 2, significant differences were observed in the number of stems and spike m^−1^ between treatments, where inoculation of isolates of GGT2 and GGT3 strongly affected the performance of these variables in wheat plants (p <0.05; **Table 4**). On the other hand, inoculation with *F. culmorum* and *R. cerealis* had an erratic performance, although slightly more aggressive in the case of *R. cerealis*. In this season, there was an increase in the number of stems and spikes in the plots inoculated with the bacterial consortium in almost all the treatments, but no significant differences were observed when comparing treatments with or without the use of the bacterial consortium. However, GGT2 +Pp showed a significant increment of 15 stems per lineal meter compared to GGT2 −Pp regardless of the high aggressiveness presented by this fungal isolate. The number of grains per spike did not show differences among the treatments (**Table 4**). There was a negative correlation between severity of infection (ISDI) and the number of spikes (Pearson: −0.34, p = 0.0308).

In terms of total biomass, no significant differences were found between +Pp and −Pp treatments for each inoculated fungus and the controls in both seasons. However, the pathogen factor resulted in differences between the treatments with fungal inoculation and the controls CNIF+Pp and CNIF−Pp in season 1 and 2 (**Table 5**). Nevertheless, average total biomass was 2.5% (7.4 g) and 6.4% (26.2 g) higher in the +Pp treatments compared to −Pp treatments in season 1 and 2, respectively. Plots inoculated with GGT2 recorded the lowest value in terms of average total biomass and grain biomass in both seasons (**Table 5**). In season 1, the GGT2 treatments were statistically different in terms of total biomass from the rest of the treatments (p = 0.05), with the exception of Rc −Pp, whereas the GGT2 treatments in season 2 were lower than all the other treatments, with the exception of GGT3 +Pp.

**Table 5:** Total and grain biomass (g) and harvest index (HI) observed in treatments of wheat cv. Pantera-INIA seed treated with (+ Pp) and without (– Pp) a consortium of three *Pseudomonas protegens* strains against crown and root rot pathogens (artificially inoculated) in two consecutive crop seasons (including 2 control treatments (CNIF) with no fungal inoculation).

	Season 1 (2016–2017)						Season 2(2017–2018)		
Treatments ^a^	Total biomass (g)		Grains biomass (g)		HI		Total biomass (g)	Grains biomass (g)	HI
CNIF + Pp	289.1 a		182.3 a		63.5 a		475.0 a	190.3 a	40.2 ab
CNIF − Pp	294.7 b		128.4 cde		43.6 bcd		433.8 ab	161.1 bc	37.4 bc
GGT2 + Pp	196.0 d		76.6 f		39.5 cd		315.3 de	100.2 d	31.7 d
GGT2 − Pp	222.9 cd		115.0 de		52.7 ab		293.3 e	102.6 d	34.8 cd
GGT3 + Pp	307.0 b		139.5 cd		45.6 bcd		395.2 bc	146.0 c	36.9 bc
GGT3 − Pp	315.7 ab		108.7 e		35.7 d		348.9 cd	119.8 d	34.5 cd
Fc + Pp	366.6 a		177.9 ab		48.4 bc		432.5 ab	157.8 bc	36.8 bc
Fc − Pp	311.0 ab		150.9 bc		48.8 bc		407.5 b	170.6 ab	42.8 a
Rc + Pp	298.5 b		152.7 abc		51.0 bc		416.3 b	156.4 bc	37.6 bc
Rc − Pp	275.9 bc		133.2 cde		48.3 bc		420.0 b	150.6 bc	35.9 bcd
**CV ^b^**	13.4		15.3		8.1		9.4	9.7	8.7
**P value ^c^**	0.0002		<0.0001		0.005		<0.0001	<0.0001	0.005

a Description of the treatments is available in **Table 1**. ^b^CV, coefficient of variation. ^c^Different letters in the same column showed significant differences among treatments according to the LSD comparison mean test (α = 0.05).

In general, grain biomass was higher in the +Pp treatments, with the exception of GGT2 in both seasons and *F. culmorum* in season 2 (**Table 5**). In this season, the control CNIF +Pp recorded the highest value in grain biomass of 182 g m^−1^, which was 138% higher than the 76.6 g m^−1^ of biomass of grains obtained by the GGT2 +Pp treatment. Values obtained in the control CNIF +Pp were between 29.6% and 15.3% higher than CNIF−Pp, and the two controls were significantly different between them in both seasons. The same situation was observed in those treatments inoculated with GGT3, where GGT3+Pp increased grain biomass in 22.1% and 17.9% when compared to the GGT3−Pp treatments in season 1 and 2, respectively. Nevertheless, Fc +Pp and Rc +Pp treatments showed higher grain biomass, especially during season 1; no significant differences were found between these treatments and those with untreated seeds (Fc −Pp and Rc−Pp) (**Table 5**).

The HI reflected the differences observed for grain biomass and total biomass. Bacterial consortium seed treatment only increased significantly the value of this index for CNIF +Pp compared to CNIF −Pp (45% more; p = 0.005) in season 1 (**Table 5**). In addition, CNIF +Pp had a higher and significantly different HI compared with the other treatments, with the exception of the GGT2 −Pp treatment in this season. In general, HI had no significant differences between all the fungal inoculated treatments (either treated or not treated with the *P. protegens* consortium) in both seasons (**Table 5**).

A correlation between the assessed variables was observed only in season 1, where total biomass and grains m^−1^ showed a significant negative correlation with respect to severity of infection (Pearson: −0.49, p = 0.0012, and Pearson: −0.52, p = 0.0005 respectively).

In general, the seed treatments with the consortium of *P. protegens* had no major effect on increasing grain test weight (**Table 6**). In season 1, treatments inoculated with GGT2 and GGT3 showed the lowest hectoliter weight values, presenting differences with respect to both controls and those plots inoculated with *F. culmorum* and *R. cerealis* (p < 0.0001). Both control treatments and those inoculated with *F. culmorum* and *R. cerealis* reached an average value close to 82 kg hl^−1^, with no differences between them (**Table 6**). The treatments inoculated with GGT2 and GGT3 showed no major differences between them, recording values below 79.6 kg hl^−1^. A similar situation was observed in season 2, where both GGT2 and GGT3 treatments reached the lowest hectoliter weight values (81–83 kg hl^−1^) and were significantly different from the rest of the treatments (p < 0.0001). On the other hand, the controls treatments and those inoculated with *F. culmorum*, and *R. cerealis* showed homogeneous weight of around 85 kg hl^1^. Regarding thousand grain weight of wheat (g), values ranged between 50 and 53 g in both seasons, so that the seed treatments with the bacterial consortium and pathogens inoculated had no effect on this parameter (data not shown).

**Table 6:** Grain test weight (kg hl^−1^) and grain yield averages (ton ha^−1^) observed in treatments of wheat cv. Pantera-INIA seed treated with (+ Pp) and without (– Pp) a consortium of three *Pseudomonas protegens* strains against crown and root rot pathogens (artificially inoculated) in two consecutive crop seasons (including 2 control treatments (CNIF) with no fungal inoculation).

	Season 1 (2016–2017)		Season 2 (2017–2018)	
Treatments ^a^	Grain test weight (kg hl^−1^)	Grain yield (ton ha^−1^)	Grain test weight (kg hl^−1^)	Grain yield (ton ha^−1^)
CNIF + Pp	82.0 a	10.5 ab	85.2 a	9.9 ab
CNIF − Pp	81.2 a	8.6 cd	85.1 a	10.4 a
GGT2 + Pp	77.6 c	7.2 ef	81.6 c	5.3 f
GGT2 − Pp	78.5 bc	5.5 g	83.1 b	5.9 ef
GGT3 + Pp	79.6 b	8.0 de	83.1 b	6.7 d
GGT3 − Pp	79.5 b	6.8 f	83.0 b	6.6 de
FC + Pp	81.8 a	10.7 a	85.5 a	9.5 bc
FC − Pp	81.1 a	9.2 c	85.3 a	10.3 a
RC + Pp	82.1 a	9.5 bc	85.5 a	9.8 ab
RC − Pp	81.1 a	8.8 cd	85.4 a	9.2 c
**CV ^b^**	1.05	8.65	0.92	5.99
**P value ^c^**	< 0.0001	< 0.0001	< 0.0001	< 0.0001

a Description of the treatments is available in **Table 1**. ^b^CV, coefficient of variation. ^c^Different letters in the same column showed significant differences among treatments according to the LSD comparison mean test (α = 0.05).

In terms of grain yield (ton ha^−1^), GGT2−Pp and GGT3−Pp recorded the lowest values, with values 35% lower than the control CNIF +Pp in season 1. The use of the consortium of *P. protegens* strains resulted in significant increases of 1.7 ton ha^−1^ and 1.2 ton ha^−1^ for plots inoculated with GGT2 (31% increase) and GGT3 (17.6% increase), respectively (p > 0.05). In season 1, plots inoculated with *F. culmorum* had the highest yields; Fc +Pp recorded a significant increase in yield when compared to Fc −Pp, reaching similar values to those recorded by CNIF + Pp. In fact, Fc +Pp and CNIF + Pp achieved yields 20% higher than the rest of the treatments, with an average of 10.6 ton ha^−1^. In case of inoculation with *R. cerealis*, no differences were found between Rc +Pp and Rc −Pp or with respect to the controls, indicating that this fungus had no effect on grain yield. In addition, a negative and significant correlation was observed between grain yield and the severity of infection and visual symptoms observed in the plots (AUDPC) (Pearson: −0.37, p = 0.0183 y Pearson: −0.87, p< 0.0001, respectively). On the other hand, positive correlations were observed between final yield and plant height (Pearson: 0.74, p <0.0001), number of stems m^−1^ (Pearson: 0.57, p < 0.0001), spikes m^−1^ (Pearson: 0.59, 0.0001), total biomass (Pearson: 0.69, p < 0.0001), biomass of grains (Pearson: 0.72, p < 0.0001), HI (Pearson: 0.40, p = 0.0103), and thousand grain weight (Pearson: 0.43, p = 0.0059).

In season 2, the addition of the bacterial consortium caused no effect on productivity, and differences between seeds treated and those untreated with the antagonistic bacteria were only found in the treatments inoculated with *R. cerealis* (P <0.01). In this sense, the Rc +Pp treatment showed an increase of 6.5% in the final productivity. Moreover, a scarce 1.5% yield increase was observed in those plots inoculated with GGT3 (p < 0.05) (**Table 6**). The two isolates of *G. graminis* var. *tritici* resulted in the highest yield losses, with an average of 6.1 ton ha^−1^ and showing significant differences with the rest of the treatments (p > 0.05; **Table 6**). The treatments inoculated with *R. cerealis* and *F. culmorum* showed no major effect on yield and only the inoculation with *Rhizoctonia* without bacterial seed treatment was different from the controls (CNIF +Pp and CNIF −Pp), which showed no differences between them. In addition, significant negative correlations were observed between final yield and incidence and severity of infection (Pearson: −0.73, p <0.0001 and Pearson: −0.75, p <0.0001, respectively). On the other hand, positive correlations were observed respect to yield components, such as number of stems m-1 (Pearson: 0.43; p = 0.0051), spikes m-1 (Pearson: 0.51; p = 0.0007), total biomass (Pearson: 0.73, p <0.0001), grain biomass (Pearson: 0.72, p < 0.0001), and HI (Pearson: 0.32, p = 0.0414).

## Discussion

Pathogens associated with crown and root rot diseases of wheat cause considerable yield losses in Chile. At present, there are not efficient control methods for these plant pathogens (De Coninck et al., 2015; Vera et al., 2019). The use of fungicide seed treatments is a common practice worldwide that has achieved certain effectiveness for the control of these phytopathogens (Vera et al., 2014; Vera et al., 2019). Therefore, the antagonistic effect of a consortium of three Chilean strains of *P. protegens* on important crown and root rot pathogens, such as *G. graminis* var. *tritici*, *F. culmorum* and *R. cerealis*, was evaluated on spring wheat during two consecutive seasons under field conditions. The seed treatment at a concentration of 10^8^ CFU g^−1^ seeds of the consortium of *P. protegens* plus carboxymethyl cellulose reached a population density of approximately 10^5^ CFU g^−1^ roots in the anthesis state (Z6) in both seasons. This value is in accordance with the minimum population density that has been reported in the wheat rhizosphere as the required to trigger the phenomenon of take-all decline in suppressive soils (Kwak et al., 2009; Yang et al., 2011). The 2.0 Log reduction in bacterial population in the 24-h period between seed inoculation with the bacterial consortium and seeding in the field could be explained by the drying conditions to which treated seeds were exposed under a laminar flow cabinet before seeding. Nevertheless, the quantification of bacterial populations based on the presence of the *+phlD* gene, associated to the production of the antimicrobial compound 2,4-DAPG, showed that seed inoculation markedly increased the natural concentration of 2,4-DAPG producing-bacteria in the rhizosphere in both seasons. These populations moved from Log 4.3 CFU g^−1^ average roots observed in the plots not inoculated with the *P. protegens* consortium to an average of Log 4.6 CFU g^−1^ roots in plots with bacterial consortium seed treated, reaching a maximum population of approximately Log 6.2 CFU g^−1^ roots in season 2 (season 2017–2018). This demonstrates that seed treatment is an adequate tool to favor the colonization of the rhizosphere by antagonistic bacteria such as *P. protegens*. This has also been reported for other species such as *P. fluorescens* (Fox et al., 2016; Imperiali et al., 2017).

The quantification of bacterial populations of *P. protegens* per root gram was 2.6 times lower in season 1, with a difference of about Log 5.3 CFU g^−1^ roots between the two seasons. This difference could be attributed to the climatic conditions of each crop season, where the variation in the accumulated rainfall had a difference of 320 mm, with season 2 being markedly rainier than season 1. The difference in rainfall regimes between the seasons could have affected the colonization of the antagonistic bacteria as described by Mavrodi et al. (2012a) and Mavrodi et al. (2018). The authors have reported that growth of *phlD*-producing pseudomonads present in wheat plant rhizosphere is favored under conditions of higher soil moisture compared to bacteria of the same genus, but without the presence of this gene. On the other hand, both studies indicated that phenazine-producing *Pseudomonas* are more frequent under conditions of lower water availability. This suggests that within a context of lower rainfall, that phenazine-producing *Pseudomonas* could be less affected and predominate over populations of 2,4-DAPG-producing *Pseudomonas*. Nevertheless, the increased growth and yield and reduced severity of disease observed in this study with the consortium of the Chilean *P. protegens* strains, particularly during the season with lower rainfall (season 1), suggest that wheat seed treatment with this consortium could be useful to improve productivity under dryland conditions. The influence of different genetic groups of the *phlD* gene (occurring in natural or indigenous population of rhizospheric bacteria) on the results of disease control and growth promotion was not addressed in this study, because all the inoculated *P. protegens* strains belong to the genetic group A of this gene. Studies conducted in our lab allowed determining that genotype group A is very common in southern Chile. However, genetic groups B, D, K, L, and P associated with the *phlD* gene are also found in Andisol soils (unpublished data).

The observed pathogenicity of inoculated phytopathogenic fungi determined that *G. graminis* var. *tritici* of the genetic groups 2 and 3 (GGT2 and GGT3, respectively) were the most pathogenic fungi. Under field conditions, both isolates were highly aggressive, affecting roots and first internode of wheat plants, decreasing total and grain biomass, and reducing accumulation of total and grain biomass, and grain yield. On the other hand, *R. cerealis* and *F. culmorum* presented an erratic and weak pathogenicity, showing in some cases similar values to both CNIF treatments in parameters such as disease symptoms (AUDPC and culm damages), final plant height, total and grain biomass, grain test weight, and grain yield. However, phytopathogenicity tests showed that all pathogens could be re-isolated from infected plant material, confirming that the fungi were capable of entering and establishing into the host, causing different levels of infection (data not shown).

Low phytopathogenicity could be related to the geographical origin of the isolates used in the experiments. *R. cerealis* isolate M31S and *F. culmorum* isolate F-CULM were obtained from wheat plants sampled from commercial field crops located between Araucanía and Los Lagos Regions (Moya-Elizondo et al., 2015). In this area, average rainfall fluctuates between 1,050 and 1,560 mm per year, while air and soil temperatures vary between 11°C and 13°C. The two field trials were conducted about 400 km north of this sampling site. In Chillán, the average rainfall recorded during the crop cycles was 307 mm, whereas air and soil temperatures were around 14°C. The differences in climatic conditions between the area where the isolates were sampled and the study area as well as the adaptation of the isolates to higher precipitation regimes and lower air and soil temperatures could account for the reduced pathogenic capacity of them. However, drought conditions favor infection by *Fusarium* (Moya-Elizondo, 2013). Therefore, drier conditions in season 1 may also explain the higher rates of AUDPC, incidence of symptomatic culms, and damage in the first internode and roots observed in the treatments inoculated with *F. culmorum*. In the case of *R. cerealis*, the results were also contradictory since this wheat pathogen is commonly observed causing problems in geographic areas with annual rainfall lower than 400 mm, as is the case of the Pacific Northwest, USA (Yin et al., 2013; Weller et al., 2016). In our research, plants presented more visual symptoms of the disease (AUDPC) in plots inoculated with *R. cerealis* in season 1, which markedly had lower rainfall than season 2. In this sense, soil moisture level at the time in which a given pathogen is most highly aggressive can also explain the results obtained, particularly because high rainfall was concentrated at plant emergence (August and September) and one or two irrigation events were conducted at the end of tillering or anthesis (October and November). Such water availability conditions could have influenced the aggressiveness of these fungi. In addition, no greater effects of these fungi were observed at crop emergence, which would indicate that these pathogens are either weak or more aggressive depending on the environmental conditions. For example, *F. culmorum* causes most pre and post-emergence death of seedlings in dry soil with increasing temperature, while smallest number of dead plants or surviving plants with lesions occurs under wet soil and low temperatures (Moya-Elizondo, 2013), which were conditions observed in both seasons, but particularly notorious in season 1. If isolates of *F. culmorum* and *R. cerealis* are weak pathogens, further research is required to determine levels of fungal pathogenicity with artificial inoculation of pathogens in the field. Screening through pot trials with artificial inoculation of the isolates in the soil substrate prior to seeding would allow evaluating and determining their levels of aggressiveness.

Even though bacterial populations were lower in season 1, the beneficial effects of the bacterial consortium of *P. protegens* on the vegetative development and productivity of wheat inoculated with root and crown pathogens were more evident than in season 2. In season 1, there was a marked decrease of aerial symptoms associated with crown and root rots pathogens, as well as a significant increase in the number of stems and spikes m^−1^, biomass accumulation, and grain yield. These results are coincident with those reported by Rubin et al. (2017), who have indicated that the effectiveness of PGPR (plant grow promoting rhizobacteria) is greater under conditions of lower soil moisture. On the other hand, Mäder et al. (2011) reported an increase of 31% in grain yield when wheat seeds were artificially inoculated with two strains of *P. fluorescens* in field experiments. In this sense, the results of our study on spring wheat are in agreement with the previously described findings, considering that artificial inoculation with a consortium of Chilean *P. protegens* strains resulted in increases of 31% in yield, 18% in total biomass, and 42% in grain biomass.

The results obtained in the present study show that the consortium of the three Chilean *P. protegens* strains have growth promoting activity as that reported for PGPR microorganisms. PGPR organisms have positive effects on plants by using direct mechanisms such as production of growth regulators, nitrogen fixation, and phosphorous (P) biosolubilization, whereas indirect mechanisms are related to biocontrol on phytopathogens through the production of antimicrobial compounds, lytic enzymes, competition for resources, niche occupation (Ramette et al., 2011; Tabassum et al., 2017; Sahu et al., 2018), and induction of resistance (Maketon et al., 2012). *Pseudomonas protegens* is characterized by the production of the antimicrobial compounds 2,4-DAPG, pyoluteorin and pyrrolnitrin, whose genes are present in the strains Ca6, Ca10, and ChB7 used in this study. In addition, the ability of these strains to solubilize P and produce IAA has also been determined in our laboratory. All the bacterial strains studied show antagonistic activity against *G. graminis* var. *tritici* and 1,3-β-glucanase activity under *in vitro* conditions, with strains Ca6 solubilize P at high levels (120 mg P L^−1^), while ChB7 and Ca10 have shown high biosurfactant production activity and IAA production (2,1–3,1 mg IAA L^−1^, respectively) (unpublished data). These differential characteristics among the Chilean *P. protegens* strains used as a consortium seed treatment in this research would explain the reduction observed in the symptoms associated with crown and root rot and their impact on growth and yield in spring wheat. Improved root colonization, reduced damage caused by fungal pathogens, and the positive effect on development and productivity of the wheat plant achieved by the inoculation of the three strains of *P. protegens* could have practical implications for the future use of these in wheat crops located in geographical areas with low rainfall. The agricultural production in Andisol soils between Ñuble (36°37’00’’S 71°57’00’’W) and Los Lagos regions (41°28’18’’ S 72°56’12’’W) in Chile is mainly characterized by being conducted under dryland conditions, in many cases associated with small farmers (Durán et al., 2017). Moreover, the genera *Gaeumannomyces*, *Fusarium*, and *Rhizoctonia* are usually found in this type of soil (Moya-Elizondo et al., 2015). Andisols are soils of volcanic origin and they are very rare in the world, representing less than 1% of the cultivable soils worldwide (Delmelle et al., 2015). An Andisol soil is characterized by a high P fixation capacity, high content of organic matter (OM), and high levels of acidity that trigger phytotoxicity by aluminum and manganese (Borie et al., 2010). Therefore, the use of bacteria that promote P solubilization and root growth is a good tool to improve the productivity of this type of soils. This is even more relevant in a scenario of climate change, considering that the use of plant growth promoting microorganisms can improve wheat productivity under condition of water stress in plants. In Chile, as in most countries, climate change is expected to have significant impacts on agricultural production, mainly due to changes in precipitation regimes. In this sense, the steady decline of precipitation recorded in Central Chile over the last decade, with rainfall deficits between 25% and 45%, has been referred to as “mega drought” (Garreaud et al., 2017). Moreover, according to models of future simulation, it is estimated that wheat yield in Chile will decrease between 15% and 20% by 2050 (Meza and Silva, 2009).

Spring wheat production in Andisol soils is facing risks of water deficit. The results of our study indicate that treating seeds with the consortium of the three Chilean strains of *P. protegens* can help improve wheat crops as its use has a biocontrol effect on different crown and root rot pathogens and is capable of promoting significant growth and yield under conditions of lower rainfall. Further studies need to be conducted to evaluate if this consortium of *P. protegens* is also efficient in other types of soils, particularly because their beneficial effects were observed when there were smaller indigenous bacterial populations and under conditions of lower soil moisture.

In season 2, a lower control and growth promoting effect was observed when wheat seeds were treated with the bacterial consortium, although greater populations of *P. protegens* were observed in the rhizosphere of spring wheat plants. Clearly, conditions of higher rainfall and soil moisture could have influenced the disease progress caused by the inoculated pathogens, but it does not explain why the bacterial seed treatment did not show marked differences in the development and productivity of wheat plant. This could be explained by the energy and/or metabolic costs associated with triggering the processes of inducing plant resistance under the presence of beneficial microorganisms. In this context, several authors consider that plants under infective episodes can activate induced resistance phenomena (Denancé et al., 2013; Vos et al., 2013). However, in order to achieve this, defense mechanisms are preferably allocated in detriment of other physiological processes, resulting in lower growth and development. The induction of resistance in wheat plants by microorganisms such as *P. fluorescens* has been related to the production of 2,4-DAPG (Maketon et al., 2012; Sahu et al., 2018). The *phlD* gene associated with the production of 2,4-DAPG is present in the *P. protegens* strains evaluated in this study, which indicates that high populations of bacteria could produce this compound in the wheat rhizosphere under conditions of greater soil moisture. Therefore, if the induction of systemic resistance in the plants is promoted, this condition could have generated a metabolic cost in the wheat plant. Thus, an environmental condition that favored the soil pathogens along with higher populations of *P. protegens* could have resulted in this phenomenon of induced resistance and increased the metabolic cost in plants. This hypothesis can be partially confirmed if we consider the results for *F. culmorum* seed treated *P. protegens* in season 2, since an average of 8% decrease in the accumulation of grain biomass and grain yield was recorded (p < 0.05). In addition, although not significant, there was a reduction in grain production for CNIF +Pp and GGT2 +Pp in season 2, which could support the suggested hypothesis. On the other hand, the increased damage observed in the first internode expressed as a discoloration in the culm for GGT2 seed treated with the bacterial consortium (+Pp) compared to its counterpart GGT2 −Pp, could be explained by a hypersensitivity response induced by the high colonization of *phlD*-producing bacteria in the roots observed in season 2. A hypersensitivity response can cause localized discoloration in the plant tissue, being part of a process associated with the triggering of induced resistance phenomena (Denancé et al., 2013; Vos et al., 2013).

The results obtained in this research suggest that seed treatments with PGPR microorganisms can be effectively used to stimulate plant growth and protect wheat crops against several phytopathogens that cause crown and root rot disease in spring wheat. This is particularly valid for the conditions of Andisols, which are present along southern Chile, and a scenario of climate change, where drought periods are expected to become more frequent and intense. Therefore, the use of a consortium of beneficial bacteria such as *P. protegens* becomes relevant and may become a possible sustainable strategy to reduce the negative impact of biotic and abiotic factors on wheat crops in this area.

## Data Availability Statement

The datasets generated for this study are available on request to the corresponding author.

## Author Contributions 

MC: conceptualization, data curation, formal analysis, investigation, methodology, writing—original draft, writing—review and editing. RM: conceptualization, funding acquisition, project administration, resources, supervision, writing—review and editing. BR: molecular investigation, writing—original draft. MV: conceptualization, methodology, supervision, writing—original draft. CV: project administration, resources, investigation. EM-E: conceptualization, funding acquisition, formal analysis, investigation, methodology, project administration, resources, supervision, writing—review and editing.

## Funding

This study was funded by the Phytopathology Laboratory of the Faculty of Agronomy of the Universidad de Concepción and the Laboratory of Plant Pathology of Cereals of the National Agricultural Research Institute INIA-Quilamapu.

## Conflict of Interest

The authors declare that the research was conducted in the absence of any commercial or financial relationships that could be construed as a potential conflict of interest.
